# Allelic imbalance of the *TGFβR1* is not a major contributor to the genetic predisposition to colorectal cancer

**DOI:** 10.1038/bjc.2011.124

**Published:** 2011-04-26

**Authors:** C Abadie, A Killian, J Tinat, M Bougeard, D Medhaoui, A-F Cailleux, S Baert-Desurmont, T Frebourg

**Affiliations:** 1 Inserm U614, Faculty of Medicine, University of Rouen, and Department of Genetics, University Hospital, Institute for Medical Research and Innovation, 22 Boulevard Gambetta, Rouen 76183, France; 2 Clinical Investigation Center, CIC 0204, Inserm and University Hospital, Institute for Medical Research and Innovation, Rouen, France

**Sir,**

We read with great interest a recently published article in your journal entitled ‘No association between germline allele-specific expression of *TGFBR1* and colorectal cancer risk in Caucasian and Ashkenazi populations’ (Seguí *et al*, 2011). Whereas an allelic expression imbalance of the transforming growth factor-*β* type 1 receptor (*TGFβR1*) gene was reported in 2008 to be a strong risk factor for colorectal cancer (CRC), with an odds ratio of 8.7 (Valle *et al*, 2008), this new study coordinated by Valle and performed on 96 CRC patients and 90 controls shows that there is no difference in blood *TGFβR1* allelic expression between CRC patients and controls. The authors indicated that the discrepancy between these two studies may probably be explained by the difference in the methods used for measuring the allelic expression, the first study being based on the SNaPshot methodology, corresponding to primer extension with dye-labelled dideoxynucleotides, the second having been performed with pyrosequencing. We would like to highlight the fact that, to determine whether or not *TGFβR1* allelic expression is a main CRC genetic factor conferring a high odds ratio, the selection both of patients and controls is a critical issue. The original report by Valle *et al* (2008) was based on CRC patients corresponding either to sporadic CRC cases or to familial cases. This new study (Seguí *et al*, 2011) was based on unselected microsatellite-stable CRC patients, without information regarding age of tumour onset or familial history, and the control population was not described. It is noteworthy that this was also a potential limitation of the additional studies on *TGFβR1* allelic imbalance in CRC published since 2008 and reporting contradictory results (Guda *et al*, 2009; Carvajal-Carmona *et al*, 2010; Pasche *et al*, 2010; Tomsic *et al*, 2010).

Therefore, we conducted a prospective case–control study on carefully selected patients and controls to clarify the real contribution of *TGFβR1* allelic imbalance in CRC. After informed consent, patients were selected according to three different criteria suggestive of an increased genetic risk for CRC: (i) CRC before 61 years of age (or high-risk adenoma before 51 years of age) with a first-degree relative presenting with CRC; (ii) CRC before 51 years of age (or high-risk adenoma before 41 years of age); or (iii) multiple primitive colorectal tumours in the same case, the first one diagnosed before 61 years of age if cancer or before 51 years of age if high-risk adenoma. Exclusion criteria were (i) Lynch syndrome defined on the basis of a germline deleterious *MMR* gene mutation or a CRC with microsatellite instability arising in a suggestive familial context; (ii) adenomatous polyposis defined as the presence of at least 10 colorectal adenomatous polyps. The controls, aged from 45 to 60 years, were CRC-free and had no familial history of CRC in their first-degree relatives. Genomic DNA was extracted from whole blood samples using the FlexiGene kit (Qiagen, Courtaboeuf, France). As high-quality RNA is a pre-requisite to perform such a study, blood samples were collected on PAXgene tubes (Qiagen), stored at −20°C and total RNA was extracted within 1 month after blood collection, using the PAXgene Blood RNA kit (Qiagen). To measure *TGFβR1* allelic expression, we used the SNaPshot methodology (Applied Biosystems, Meylan, France) described in the initial report of Valle *et al* (2008). For each patient and control, genomic DNA was first genotyped for the following four *TGFβR1* SNPs: *rs334348*, *rs334349*, *rs1590* (in total linkage disequilibrium) and *rs7871490*, to identify heterozygous individuals. After RT–PCR was performed from total RNA using oligo-dT (New England Biolabs, Ipswich, MA, USA) and reverse transcriptase SuperScript II (Invitrogen, Cergy-Pontoise, France), allelic expression of *TGFβR1* was measured, using SNaPshot, by normalising the ratio of the allelic peaks obtained on RT–PCR with the ratio of the allele peaks obtained on genomic DNA. The allelic expression was calculated from the average of the ratio obtained for the four SNPs, when individuals were informative for all these SNPs; for individuals informative only for the *rs7871490* SNP, the analysis was performed on two independent RT–PCRs. Among 175 Caucasian controls and 110 Caucasian patients genotyped for the four SNPs, we measured allelic expression of *TGFβR1* in 98 informative controls (56%) and 69 informative patients (63%). Using the SNaPshot analysis, we did not encounter any reproducibility problem: for heterozygous individuals for the four SNPs (*rs334348*, *rs334349*, *rs1590* and *rs7871490*), we obtained consistent ratio values between each SNP and, even for the *rs7871490* SNP localised in a repetitive sequence, we observed reproducible results between two independent analyses (average s.d.=0.075). Allelic expression ratios ranged between 0.82 and 1.41 (mean 1.06) in controls and between 0.77 and 1.45 (mean 1.07) in patients with the relative same compacted distribution of values in both analysed groups (see Figure 1). No significant difference in allelic expression ratios between patients and controls was observed (Mann–Whitney test *P*=0.72; Student's test *P*=0.58; permutation test *P*=0.56).

In conclusion, we confirmed by studying highly selected patients and controls that allelic imbalance of the *TGFβR1* is not a major contributor to the genetic predisposition to CRC. Therefore, measurement of *TGFβR1* allelic imbalance has no clinical utility to identify patients with high CRC genetic risk.

## Figures and Tables

**Figure 1:**
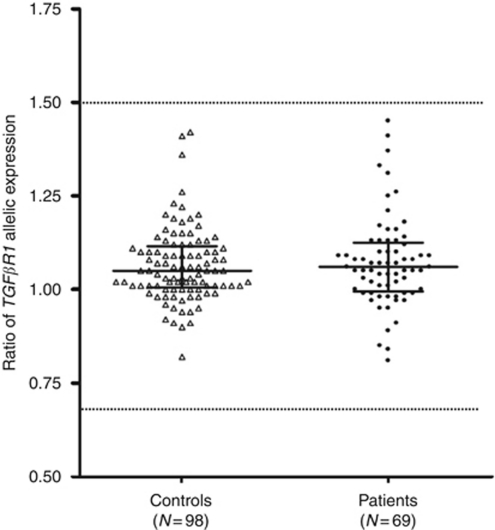
Ratios of *TGFβR1* allelic expression in 98 informative controls and 69 patients. The median and inter-quartile range values are indicated by continuous traits. The cutoff values, indicated as discontinuous lines, were those defined according to Valle *et al* (2008).
